# Orchard meadows: consumer perception and communication of a traditional agroforestry system in Germany

**DOI:** 10.1007/s10457-023-00840-4

**Published:** 2023-03-23

**Authors:** Sophia M. Philipp, Katrin Zander

**Affiliations:** Department of Agricultural and Food Marketing, Faculty of Organic Agricultural Sciences, University of Kassel, Steinstr. 19, 37213 Witzenhausen, Germany

**Keywords:** Traditional agroforestry systems, Orchard meadows, Biodiversity, Consumer preferences, Apple juice

## Abstract

Europe has a large variety of historic cultural agroforestry systems which provide numerous ecosystem services. Traditional agroforestry landscapes are characterized by a high level of biodiversity, but they lack an economic basis due to considerable time and financial effort required for cultivation, maintenance, and harvesting. Orchard meadows (OM) are a typical example for agroforestry systems. They combine large fruit trees with undercropping or livestock raising. This study investigates consumer knowledge and preferences for OM products and the possibilities of improved communication to increase consumer demand. Focus groups were conducted with German consumers. The results demonstrate that consumers have a very positive perception of OM juice in terms of taste, local production, health, and environmental benefits. In order to increase the demand for OM juice, communication with consumers needs to be improved by highlighting these positive attributes.

## Introduction

Europe has a large variety of historic cultural agroforestry landscapes. Often they combine elements of animal husbandry with grassland or arable farming and forestry (Mosquera-Losada et al. 2012). All of them provide a high diversity of ecosystem services due to large habitat variation over space and time while also supporting high levels of farmland biodiversity (Mosquera-Losada et al. 2012; Plieninger et al. 2020; Torralba et al. 2016). Besides biodiversity conservation, agroforestry systems provide benefits in carbon sequestration, soil enrichment, improving air and water quality (Jose 2009; Plieninger et al. 2020) but also form rural cultural heritage, societal and aesthetic values (Flinzberger et al. 2020).

Examples for traditional agroforestry systems in Europe are olive-, chestnut-, and cork oak-based tree cropping systems in the Mediterranean Basin (Wolpert et al. 2020) and orchard meadows (OM) in temperate Europe. OM are a widespread traditional agroforestry system present from the Atlantic coast in the west through central Europe to Hungary and Romania in the east (Forejt and Syrbe 2019; Herzog 1998). The trees in OM which are planted in wide distances, are mostly high-trunked, strong-growing and large crowned. The systems combine different ages, sizes and species of trees. Due to this diverse structure they are characterized by a high level of various ecosystem services (Forejt and Syrbe 2019; Herzog 1998; Moreno et al. 2018; Pantera et al. 2018; Tojnko et al. 2011). While OM have played an important role in agricultural cultivation in Europe in the past, nowadays the area of OM is declining, mainly due to lack of economic viability (Forejt and Syrbe 2019; Herzog 1998; Plieninger et al. 2015).

Traditional agroforestry landscape elements require management practices which are often incompatible with current agriculture management practices and go along with higher production costs (Antrop 2005). Although farmers might be aware of the enviromental benefits provided by agroforestry systems, high costs of implementation, lack of financial incentives and missing marketing strategies prevent them from the adoption (Tsonkova et al. 2018; Sollen-Norrlin et al. 2020). That is why the preservation of traditional landscapes with high level of ecosystem services needs specific support, either by policy or by markets (Flinzberger et al. 2020; Zander und Waibel 2005).

In line with changing overall societal values and with growing importance of sustainability there is an increasing interest of consumers in environmental and social effects of food (Angus et al. 2022). Sustainable and ethical consumption means to select products according to the additional values of products and production processes, such as the provision of ecosystem services, animal welfare, or social issues (Solomon et al. 2019; Stampa and Zander 2022). In this regard, understanding consumer’s perceptions of and their preferences for these additional values is decisive for the development of promising marketing strategies for agroforestry products (Gao et al. 2014).

Using the example of OM, this paper aims to examines consumers’ perception and attitudes for this agroforestry system. Based on this, criteria for consumers' purchasing decisions for products from OM and promising communication of OM products is identified.

This article is divided into six sections. Following the introduction, the second section gives an overview of OM with respect to their relevance in providing ecosystem services and the concept of local surcharge initiative as marketing and conservation organizations. Ethical consumer behavior is also discussed. The third section describes the qualitative study design. The results are presented in the fourth section which is followed by the discussion in the fifth section. The article closes with some conclusions in the sixth section.

## State of the art

### Orchard meadows, products, and marketing approaches

OM are an agroforestry system which combines fruit growing with undercropping or livestock raising. It contrasts strongly with modern intensive fruit orcharding, mostly characterized by low-trunk, densely cultivated trees in monocultures with high external input (Flinzberger et al. 2020; Herzog 1998; Zander and Waibel 2005). OM provide a high level of ecosystem services including cultural, regulating and provisioning services. The traditional landscape was most widespread from the eighteenth to the middle of the twentieth century and was used for the subsistence of rural people. It emerged from peasant cultivation and by consecutive reorganizations form an integral part of local cultural heritage. Due to this, it is related to recreation, social relations, aesthetics, and sense of place (cultural services) (Herzog 1998; Plieninger et al. 2013). For regulating ecosystem services, OM provide pollination, climate regulation, flood mitigation, erosion control and water purification (Herzog 1998). Due to their species and genetic diversity OM have potentially a high resilience towards climate change (Fischer 2007; Forejt and Syrbe 2019). The fruit from OM is mainly used in juice production but also for direct consumption or spirits (provisioning services) (Herzog 1998).

The OM system has suffered a sharp decline since the 1950s (Herzog 1998) which continues until today (Forejt and Syrbe 2019; Plieninger et al. 2015). Along with the expansion of settlements, the main cause of the dwindling populations is the lack of profitability for cultivators (Plieninger et al. 2015). The so-called ‘Aufpreisinitiativen’ (surcharge initiatives) try to establish an economic incentive for cultivators (Flinzberger et al. 2020). Their objective is to conserve locally important OM, considering their value for wildlife, landscape, and the environment. The initiatives set standards for cultivation and establish quality assurance systems for production and processing. Only high-trunked trees are allowed to be planted and the application of pesticides and synthetic fertilizers is excluded (Keech 2017; NABU 2018). The idea behind these marketing projects is to reward the extra effort for environmentally friendly production by a higher payment (‘surcharge’) for cider fruit (NABU 2018).

Naturally cloudy direct apple juice (unfiltered), is the main product of the initiatives (NABU 2018). Orchardists and other tree owners, including private people, supply their fruit seasonally to juice press houses which produce juice for the initiatives. The juice presses are partly responsible for distribution and marketing, while the initiatives are supporting them, e.g. in the search for new sales outlets, in press relations work, information booths and tasting events. In other concepts, OM initiatives manage marketing themselves, along with the organization and financial transactions (Keech 2017). Not all the fruit is sold at a premium price, since the amount of fruit delivered exceeds the sales volume. Especially smaller projects limit the accepted delivered quantity of cider fruit as they cannot sell the whole juice amount due to a lack of time and marketing knowledge. Due to these limits, only a minor share of the OM initiatives achieves the current a cost-covering producer price (NABU 2018).

### Ethical consumer behavior and communication in the food sector

The term ‘ethical consumers’ refers to a consumer segment which is concerned with ethical issues and is willing to adapt their purchasing decisions accordingly. Several studies prove consumers’ interest in ethical values and the increasing relevance of ethical consumerism also in food purchases. Ethical consumerism is the market answer to the need to increase sustainability in agricultural production by remunerating for ecosystem services via higher product prices. It is a growing trend (Angus et al. 2022; Klink et al. 2014; Langen 2013; Lusk and Briggeman 2009; Miele and Evans 2010; Newholm and Shaw 2007; Solomon et al. 2019; Vermeir and Verbeke 2006; Zander and Hamm 2010) which is reflected e.g. by the impressive growth rates of organic products in recent years (Schaack et al. 2022) or by the increasing consumer interest in animal friendly husbandry (Weible et al. 2016).

From a marketing perspective, ethical products offer a ‘plus’ compared to common products. This added value helps product differentiation (Solomon et al. 2019), implies an additional benefit to ethically oriented consumers and may also result in a higher willingness to pay. The additional benefit of the purchase of an ethical or sustainable product is the feeling of having done something good for oneself, for others and for the environment (Karampournioti 2020; Lee and Hwang 2016; Zander and Hamm 2010; Zander et al. 2013).

The relationship between ethical concerns of people and their role as individual consumers is not intuitive (Schaffner et al. 2015), because the benefits of environmentally friendly products are usually long-term. Most consumers are not overly concerned about the future consequences of their current purchasing behavior (Davari and Strutton 2014), or its effect on ecosystem services and benefits emerging from agroforestry systems (Flinzberger et al. 2020). Consumers also often link environmentally friendly purchasing behavior with higher costs in terms of money, time, and effort (Lindberg and Steg 2007).

Generally, decisions on food purchase are expected to follow habitual or limited decision making. Individual internal processes such as knowledge, learning, involvement, perceptions, preferences, intentions, valuations, motivations, attitudes, values, and emotions are all decisive for consumer behavior. Knowledge and information are of high importance for consumers’ purchasing decisions. Information search as part of purchase decision is a supplement to existing knowledge regarding the product concerned. Thus, decision-making and information search are closely related (Evans et al. 2006; Solomon et al. 2019).

The relationship between ethical concerns of people and their role as individual consumers is not intuitive (Schaffner et al. 2015), because the benefits of environmentally friendly products are usually long-term. Most consumers are not overly concerned about the future consequences of their current purchasing behavior (Davari and Strutton 2014), or its effect on ecosystem services and benefits emerging from agroforestry systems (Flinzberger et al. 2020). Consumers also often link environmentally friendly purchasing behavior with higher costs in terms of money, time, and effort (Lindenberg and Steg 2007).

Generally, decisions on food purchase are expected to follow habitual, or limited decision making (Evans et al. 2006; Solomon et al. 2019). Individual internal processes such as knowledge, learning, involvement, perceptions, preferences, intentions, valuations, motivations, attitudes, values, and emotions are all decisive for consumer behavior. Knowledge and information are of high importance for consumers’ purchasing decisions. Information search as part of purchase decision is a supplement to existing knowledge regarding the product concerned. Thus, decision-making and information search are closely related (Solomon et al. 2019).

Ethical consumerism is a complex decision-making process in food consumption (Carrington et al. 2010). Typically, ethical consumers are highly involved in their ethical purchase decisions. They can be expected to perform extensive information search when aspects of a product are relevant and noticeable for them. Thus, information should be provided in a manner which takes consumers’ selective ways of information search into account (Devinney et al. 2010; Zander and Hamm 2010). However, pure information strategies are not useful; rather, attention should be paid to adequate and relevant information that attracts attention (Schaffner et al. 2015).

Ethical product attributes are so-called credence attributes (Lee and Hwang 2016), which means that their existence cannot be verified by consumers before or after purchasing. In food purchases, the product packaging is very important for information provision. Product packaging and its design aims to differentiate goods from others and highlights product attributes, which are important for the purchasing decision. It presents the product to the exterior and is therefore intended to encourage the customer to buy it. It is particularly important at the point of sale when a product is first perceived because consumers judge the quality of a product primarily from its external appearance. Here, attracting attention and at the same time meeting the different information needs of diverse target groups is essential. Easy-to-read information that allows purchasers to classify the product at first glance is especially relevant for fast shopping, where consumers scan products only briefly. In this case, a concise message, i.e., key information is particularly important because consumers perceive only a fraction of the information available, when assessing the product (Schmid et al. 2005; Solomon et al. 2019).

## Materials and methods

### Focus groups as an explorative method

The strength of qualitative research consists of its explorative character and the possibilities of identifying salient attributes. Focus groups are planned and structured discussions (Finch and Lewis 2003) following a variable set of guiding questions which allow adapting to the natural flow of the discussion. The participants discursively exchange arguments and encourage each other to make frank contributions. The advantage of discussing in groups is to get an initial overview of the range of perspectives. Contrary to individual interviews, the focus group approach assumes that individual opinions are influenced by context and are therefore closer to everyday conversations. Focus groups support the reconstruction of experiences, interpretations, and individual factors relevant to action and decision-making (Krueger and Casey 2015; Morgan 1997).

Usually focus groups are face to face meetings. Due to increasing utilization of digital tools, online research is gaining importance. First, the analysis of chats and online forums received attention (Krueger and Casey 2015; Morgan 1997). With the catalyst of the global pandemic (COVID-19) more researchers utilized online conferences in terms of qualitative research. Advantages emerge in a more diverse composition of the groups from different areas and across geographical borders. One disadvantage concerns the exclusion of participants with limited internet access or bandwidth (Lobe et al. 2020). Since there has been little research on consumer perception and knowledge regarding OM, focus groups were chosen to collect explorative data and to get an overview of the variety of opinions. By using online focus groups, the specific restrictions during the pandemic could be met, including at the same time participants from different regions.

### Sampling

Usually two to five focus groups, depending on the objectives and the available resources, are sufficient to get insight into a subject (Krueger and Casey 2015; Morgan 1997). The suitable number of focus groups depend on the topic and on the diversity of the participants’ opinions. In online focus groups, it is important to ensure that the number of group members allows for an exchange of ideas and the visibility of all participants. Thus, a smaller group size, between 4 to 8 participants, should be preferred compared to face-to-face formats. A heterogeneous composition of the groups creates a more inclusive discussion characterized by multifaceted arguments (Krueger and Casey 2015; Lobe et al. 2020; Morgan 1997).

To receive a wide range of different opinions, central recruitment criteria were defined in advance. In this study, five focus group discussions were conducted with a total of 32 participants; each consisted of six to eight. The focus groups were conducted in September and October 2021, and lasted approximately 90 min. They were held online via the videoconferencing tool Webex. A pre-test was carried out in advance to examine the suitability of the set of guidelines.

The sample covers consumers who buy apple juice. The exclusion criteria ‘no employment in fruit growing, agriculture or market research’ were binding. The following socio-economic criteria for each focus group were specified:

- Age: 50% between 20 and 45 years of age, 50% between 46 and 75 years of age
- Gender: 50% male and 50% female
- Occupation: more than 75% employed (full or part time).

The participants were recruited by snowball sampling. There was a call for participation with the chance of winning an enjoyment package. Sports clubs and mailings lists were requested without reference to the topic. A majority of the participants resided in different areas of Baden-Württemberg, Germany, and an additional focus group was conducted with participants from outside this federal state from Germany.

### Study design

A semi-structured discussion guide with main- and sub-themes and corresponding questions was prepared in advance. The discussion started with a short introduction and by asking the participants for their favorite juice. Following this, they were asked which criteria were relevant when buying apple juice. As a transition to the main topic, participants were asked to imagine they were standing in front of the shelf of beverages in a grocery store and wanted to buy an apple juice. Two bottles of apple juice were shown to provide a stimulus for discussion about general purchasing criteria of apple juice and to explore initial awareness of OM juice. The participants were asked why they would choose a particular juice, and about their assumption of how the apples were grown and where they came from.

The moderator led over to the main topic of the discussion with the question: ‘What do you understand by OM?’ Participants were asked to discuss their expectations they attributed to OM. An input from the moderator included a short presentation of the OM characteristics compared to intensive apple production (see Table 1). By providing this information, all participants achieved comparable knowledge levels. They discussed their associations with the information displayed and whether the information provided affected their juice choice. By providing this information, all participants achieved comparable knowledge levels. They discussed their associations with the information displayed and whether the information provided affected their juice choice.

**Table 1:** Information given to participants about OM and intensive fruit growing in comparison, based on literature [Image copyright: Zander; Kreuzberg]

Characteristics	Orchard meadows	Intensive fruit growing
		
Cultivation method	Scattered fruit trees with broad distance; rare use of insecticides, fungicides, herbicides, and fertilizers	Monoculture low-stemmed trees use of insecticides, fungicides, herbicides, and fertilizers
Biodiversity	Very high different species, varieties, sizes, age; habitat for many different (rare) animal and plant species	Low
Utilization period	50–150 years	10–20 years
Work effort/kg apples	High	Medium
Use of apples	Almost all apples are used for juice production	Dessert fruit, rejects are used for juice production
Taste	Diverse	Uniform

In the following, the most convincing argument for buying and drinking OM apple juice and potential contra arguments were asked for. Finally, consumer communication of OM was discussed by asking ‘How should a label from a bottle of OM juice look like to you?’. The participants were asked to describe appealing images and messages. To incentivize the discussion, six very different existing labels of OM apple juice were presented two by two.

The focus groups were conducted in September and October 2021, lasted approximately 90 min, and were moderated by the first author. They were held online via the videoconferencing tool Webex. A pre-test was carried out in advance to examine the suitability of the set of guidelines.

## Results

### Knowledge and perception of orchard meadows

Few discussants had a direct connection to orchard meadows (OM), e.g. through relatives or friends still owning trees and meadows. In particular, participants from southern Germany were familiar with OM along roads or near villages. Discussants from central and northern Germany were less aware of OM from their near surroundings. They were only able to report on a few specific meadows or trees in their vicinity. The ‘Alte Land’ as well as ‘Lake Constance’ were named repeatedly as fruit-growing areas in all focus groups. These regions are important fruit growing areas, characterized by intensive tree plantations. OM are not typical in these regions. This reflects the difficulties participants had in defining OM and differentiating them from intensive fruit production.

When asked for their associations with OM, it became obvious that all participants had heard the term before. Mostly vague, but positive associations were related to it.

*I don't know exactly why, but I think that OM is kind of great.* (FG5, P5A)

Regarding more detailed descriptions of this method of fruit cultivation, several assumptions were made. Participants guessed OM to be scattered trees in meadows without any recognizable order, standing in a ‘jumble’. Meadows were considered as an important element of the system. The trees were considered to be old, not trimmed, widely spaced with different sizes. Participants mainly related apples with OM and to a much lesser extent, other types of fruit, e.g., pears and cherries. The diversity of varieties, e.g., different apple varieties, was also discussed, but to a lesser extent. The image of unused fruit was prevalent in all focus groups. This was described by the participants from their everyday observations in the landscape around them: the fruit falls from the tree but is only rarely picked up and mostly rots underneath the trees.

Repeatedly, the contrast between the perfectly shaped apples from standardized trees in intensive plantations and the natural ones from OM was mentioned. While OM was considered to be mostly small-scale and natural, intensive orcharding was associated with large-scale production. Participants described their impression of an intensive fruit plantation, in which standardized, trimmed and small trees are arranged in rows. The concept of ‘natural’ cultivation in OM was stressed in contrast to this industrial mass production. In general, participants perceived OM as something traditional and original, originating from the past.

*Somehow natural. Not cultivated in a plantation, planted and managed according to industrial standards, but primal, indigenous.* (FG5 P5D)

Participants disagreed regarding management and economic effort. Often the perception was stated that the meadows and trees are not or hardly cared for. The majority of the participants believed it was natural management, in which trees are allowed to grow as they want.

*They don't specifically take care of it by driving through, trimming, pruning, applying pesticides or mulching, or whatever else is done to an organic apple. So, they just stand there.* (FG2, P2D)

Few discussants pointed out that a certain amount of maintenance is necessary to prevent trees from becoming overgrown by shrubs, etc. They referred to mulching, mowing by tractor, or grazing. In contrast to intensive orchards with a high level of machinery and resources use, participants expect hardly any interference in the natural growth in OM. They were not sure about the use of fertilizers and of pesticides. The majority supposed that neither spraying nor fertilizing occurs in OM.

Harvesting was perceived to be hard work. Some participants suspected that due to low profitability, the fruits were not harvested and processed. This was seen as the reason for the absence of new plantations.

*I would say that OM is not to bring totally much yield. Rather, it is simply permitted to grow. And if an apple emerges, that's fine. But that's not a necessity.* (FG5, P5E)

This was accompanied by the assumption that OM is mostly unmanaged for economic reasons and not in a professional agricultural context. Fruits from OM were not perceived to be an important raw material provider for the apple juice industry. In the case of OM juice, the participants tended to expect local production, which was why they expected the juice to be more difficult to obtain. Juice in glass bottles was rather presumed to be originating from OM while juice filled in tetrapacks^®^ was imagined to be from intensive apple plantations and produced from concentrate by huge companies.

The important role of OM for biodiversity and as habitats for different animals and plants was mentioned few times, especially by participants who knew OM from their immediate surroundings. When mentioned, mainly birds and insects were addressed. Thus, biodiversity apart from the aforementioned statements about fruit diversity was hardly a topic in the unprompted associations.

### Arguments for buying orchard meadows juice

Before discussing arguments specifically for buying OM apple juice, participants were asked for their general purchase criteria when buying apple juice. Frequently mentioned items were:

- Local origin
- Type of packaging: glass bottles
- Taste: less sweet
- Direct juice: not from concentrate
- Turbidity: naturally cloudy

The majority of the respondents paid attention to the fact that the juice they bought was local or that the producer was local and familiar to them. Even though glass bottles are occasionally not purchased because of their weight, this type of packaging was preferred due to environmental concerns, taste and the association with higher quality and local origin.

*The apples coming from abroad will probably not end up in glass bottles, but in tetrapacks*^*®*^ *or other containers. I think most of the glass bottles come from Germany*. (FG2, P2D)

Participants tended to link glass bottles with less added sugar than juices in tetrapacks^®^. Taste was also a frequently mentioned purchase criterion. A slightly acidic, fresh and less sweet taste was preferred. The respondents tended to expect this in direct and naturally cloudy juice and also in local juice. Some of the participants linked the aspect ‘no added sugar’ with direct juice. Direct juice was also favored because of the assumption of less intense processing and fewer additives in contrast to juice made from concentrate.

*The big companies* [...] *will buy it as a concentrate and then somehow mix it together.* (FG4, P4A)

Organic production and product price played a minor role among respondents. Even less respondents indicated to pay attention to the fact that the juice came from OM when buying juice.

Up to this stage of the discussion, the answers of the participants were based purely on their own knowledge and the interaction among them. In the following, the discussion is based on the effect of the provided information on differences in fruit growing methods (see Table 1). After describing the two orcharding methods, participants saw the potential of better information for changed purchasing behavior in favor of OM juice:

*On the question of whether it matters that it says "Streuobst" or not: I think that without the background information that I have now, it wouldn't matter so much to me. But now, after I can learn more from this group, I would probably pay more attention in the future to whether it says ‘Streuobst’ or not.* (FG3, P3A)

Four main arguments for purchasing OM juice emerged in the discussion about the characteristics and differences between juice from OM compared to intensive orcharding:

- Taste: not uniform, intensive
- Naturalness: reduction of synthetic pesticides; traditional way of fruit growing
- Local origin: reduction of transport distances
- Biodiversity: preservation of traditional fruit varieties; species-rich fauna and flora

Participants welcomed the fact that the juice was composed of many different varieties of apples. Obviously, a more intense and interesting taste was the most convincing argument. Participants assumed this to be a consequence of the diversity of varieties. One participant also mentioned a less sweet taste than from intensively produced fruit, which again spoke in favor of buying OM juice. Also, a local origin was associated with OM juice and appreciated. Besides environmental concerns, the support of local engagement and preservation of landscape was also mentioned as an advantage of local juice.

*That it is not from just anywhere, but they come from the surrounding area, where there are lots of meadows and lots of farmers who deal with it.* (FG 1, P1C)

The local origin was not presented as being a typical attribute for OM, but frequently named in the focus groups as an important purchase argument. Both attributes—taste and local production—had been mentioned before as important aspects when buying juice in general.

It is precisely biodiversity in OM—beyond the diversity of varieties—that was another frequently cited argument in favor of OM juice. Thus, the participants stated that they would buy OM juice in order to preserve this traditional and natural form of cultivation. They considered OM to be sustainable. In this context, again the term ‘naturalness’ occurred. Here, the participants referred to the reduced use of pesticides, the more natural growth of the trees, and the reduced mechanical and chemical intervention.

*That it is just natural. It's the way it grew. It's quite rare that you still get that. Not influenced by a thousand things, that are sprayed over it and goals that are pursued with thicker apples* […]. (FG4, P4B)

### Communication and labeling

Based on several label from OM juice bottles, the participants emphasized the importance of a clear structure of the label when buying juice. It was crucial to participants that the individual elements are placed in a way which allows to obtain a quick general idea of the product. The content of the product should be easily recognizable.

*It's important for me to know what's in there.* (FG2, P2C)

Labels should contain information which is of interest to consumers and relevant for their purchasing decisions. However, overloading labels must be avoided. Respondents preferred clear delineations, and uncluttered borders or frames. The size of terms displayed on the label should be in relation to their importance. This applies to both, images and wording. Important information was considered to be apple juice, OM (Streuobst) and “100 percent fruit content”. Local origin of the fruit juice is also interesting for consumers and should be included in form of the names of local manufacturers or initiatives or a rural village. An image that refers to OM was important for the participants to easily differentiate OM juice from other apple juices.

In addition to the textual components already listed, which were frequently addressed by the participants, there were other terms that were only occasionally named as desired components. They include information referring to the protection of nature, species, and the environment. The focus group participants liked the slogan "Nature protection that tastes good for everyone" and "Fair price for growers". Also, a logo of a nature conservation association was appreciated. However, most respondents were skeptical about other logos. The organic logo was benevolently registered, but according to the respondents, it would have been sufficient to state that no pesticides were used and to refer to local or German origin. The same applied to other logos. They were perceived as positive additional features but could also be replaced by the terms already mentioned.

The perception of OM juice originating from a natural way of cultivation should be reflected in the choice of natural colors, a plain layout and reference to nature in the images. For example, an apple should look fresh, but might have a slightly imperfect shape.

Respondents often expected smaller producers or initiatives to produce OM juice which was also associated with less professionality. A local context was important to most of the participants. This was conveyed less via terms such as ‘locally’ or ‘homegrown’ but rather via the exact specification of the area of origin. Preferably, this should describe a rural area and big cities. The reference to a local OM initiative was associated with authenticity, sympathy, and included social motives. By buying a local juice, they could support local producers, initiatives, or presses. This information even compensated for an unprofessional design or missing references to OM. Few participants also mentioned that a sense of identification and familiarity would also be offered by a well-known personality, the cultivator himself, a nature conservation association on the label.

## Discussion

Consumer knowledge of the specific benefits of orchard meadows (OM), i.e. ecosystems services was rather low. This result is comparable with previous research on the role of ethical consumption for the conservation of agroforestry systems. Looking at Mediterranean agroforestry systems, Flinzberger et al. (2020) stated that consumers are not yet aware of the ecosystem services and benefits emerging from these cultivation forms. Lack of consumer knowledge has proven to be major inhibitor for environmentally friendly and ethical consumer behaviour (Carrington et al. 2010; Khai and Yabe 2015; Lee 2019; Mazzocchi et al. 2019; Naspetti and Zanoli 2021; Tu et al. 2021, Weible et al. 2016) and demonstrates the importance of consumer knowledge about production methods for environmentally friendly purchasing behavior. Having basic knowledge is one of the prerequisites for transferring attitudes into concrete behavior (Carrington et al. 2010).

OM juice fulfills almost all the product attributes consumers are paying attention to when buying apple juice: local origin, glass bottles, less sweet taste, and direct and naturally cloudy juice. Regarding both—purchase criteria for apple juice in general and stated motives for choosing OM juice—it became evident that product attributes that provide a hedonic value to the individual consumer, such as taste or health, take precedence over ethical aspects when purchasing apple juice. Taste was mentioned as an important criterion. Naturalness is reflected in the attributes ‘direct juice’ and the ‘natural cloudiness’. The higher naturalness of OM juices was perceived as an advantage for one's own health: fewer pesticides, fewer additives. The positive reputation of naturalness of food for many consumers has already been reported by several studies focusing on different agricultural products and food (Berry et al. 2017; Hüppe and Zander 2021; Meier et al. 2019; Román et al. 2017; Siegrist and Hartmann 2020; Siegrist 2008). This has also been described as the’natural-is-better’ heuristic with the main aspects of being healthier, environmentally friendly and providing better taste (Siegrist 2008).

Along with taste, health and naturalness, respondents stated ecosystem services, such as locally produced and biodiversity as reasons for purchasing OM juice. The importance of local products as strong ethical purchasing argument has been widely examined and confirmed in recent years (Vargas et al. 2021; Zander and Hamm 2010). Conservation of biodiversity played a comparatively minor role in this study focusing an OM in contrast to the results gathered by Gao et al. (2014) by studying consumer perceptions of agroforestry systems in general.

## Conclusions

Food production in agroforestry systems goes along with a high level of ecosystem services, however production costs are higher, so that production is not competitive. The example of OM shows that consumers are interested not only in the ecosystem services of the products, here apple juice, but also in the core product quality such as taste, healthiness, naturalness. The results of this study demonstrate significant challenges entailed in motivating consumers to support agroforestry systems through their purchasing actions.

The positive connotation of OM in general, of the specific environmental benefits as well as of important characteristics of the main market product from OM, which is apple juice, opens the door for higher consumer demand. Consumer communication should focus on the link between personal benefits to consumers, such as less sweet taste and health aspects and ethical attributes like environmental protection and the support of local initiatives. It is important for ethically motivated consumers to be able to access further information especially about these attributes. Given the limited space of the product label, additional possibilities for getting further information should be provided. For example, an internet address, barcode or QR code scanning via smartphone would allow consumers with a higher level of involvement and interest to search for additional information.

The idea of OM initiatives is to market the juice from OM at higher prices to consumers. The success of these initiatives is very variable and their relevance compared to still existing OM area is small. OM are still heavily declining. Against this background the research at hand showed that OM initiatives could take a leading position in transparent consumer communication and information provision. As players with local position, OM initiatives can contribute to sensitize and inform people for both the purchase of OM products and the relevance of OM in terms of providing ecosystem services. Nevertheless, improving marketing and communication strategies needs professional expertise, which is often lacking in the OM initiatives.

Given the fact that OM are providing public goods as ecosystem services which are relevant for society, political action is needed to help OM initiatives to achive the professional skills they need for successful marketing of their ‘ethical’ products by granting advice and training. In addition, production needs to be directly subsidised.

The research at hand is based on qualitative explorative research to better understand consumers perceptions and preferences. A further examination of consumers’ preferences for OM products via a quantitative analysis, including consumers’ specific preferences, attitudes and purchase behavior, can help to identify relevant target groups and to improve communication considering consumers' prior knowledge and their needs and wants.

## References

[CR1] Angus G, Westbrook A (2022) Top 10 global consumer trends 2022. Euromonitor international. https://go.euromonitor.com/white-paper-EC-2022-Top-10-Global-Consumer-Trends.html. Accessed 25 Jan 2022

[CR2] Antrop M (2005). Why landscapes of the past are important for the future. Landsc Urban Plan.

[CR3] Berry C, Burton S, Howlett E (2017). It’s only natural: the mediating impact of consumers’ attribute inferences on the relationships between product claims, perceived product healthfulness, and purchase intentions. J Acad Mark Sci.

[CR4] Carrington G, Neville M, Whitwell B (2010). Why ethical consumers don’t walk their talk: towards a framework for understanding the gap between the ethical purchase intentions and actual buying behaviour of ethical minded consumers. J Bus Ethic.

[CR5] Davari A, Strutton D (2014). Marketing mix strategies for closing the gap between green consumers’ pro-environmental beliefs and behaviors. J Strateg Mark.

[CR6] Devinney T, Auger D, Eckhardt G (2010). The myth of the ethical consumer.

[CR7] Evans G, Jamal M, Foxall A (2006). Consumer Behaviour.

[CR8] Finch J, Lewis H, Ritchie J, Lewis J (2003). Focus groups. Qualitative research practice. A guide for social science studentsand researchers.

[CR9] Fischer M (2007). Streuobst—Betreiberkonzepte und Sortenempfehlung. Erwerbs-Obstbau.

[CR10] Flinzberger L, Zinngrebe Y, Plieninger T (2020). Labelling in Mediterranean agroforestry landscapes: a delphi study on relevant sustainability indicators. Sustain Sci.

[CR11] Forejt M, Syrbe RU (2019). The current status of orchard meadows in Central Europe: multi-source area estimation in Saxony (Germany) and the Czech Republic. Morav Geogr Rep.

[CR12] Gao J, Barbieri C, Valdivia C (2014). A socio-demographic examination of the perceived benefits of agroforestry. Agrofor Syst.

[CR13] García de Jalón S, Burgess PJ, Graves A, Moreno G, McAdam J, Pottier E, Novak S, Bondesan V, Mosquera-Losada R, Crous-Durán J, Palma J, Paulo J, Oliveira T, Cirou E, Hannachi Y, Pantera A, Wartelle R, Kay S, Malignier N, Vityi A (2018). How is agroforestry perceived in Europe? An assessment of positive and negative aspects by stakeholders. Agrofor Syst.

[CR14] Herzog F (1998). Streuobst: a traditional agroforestry system as a model for agroforestry development in temperate Europe. Agrofor Syst.

[CR15] Hüppe R, Zander K (2021). Consumer perspectives on processing technologies for organic food. Foods.

[CR16] Jose S (2009). Agroforestry for ecosystem services and environmental benefits: an overview. Agrofor Syst.

[CR17] Karampournioti E (2020) Understanding consumers’ ethical decision-making process: assessment of antecedents and consequences of consumer’s explicit and implicit perception and behavior towards ethical consumption. Dissertation, Gottfried Wilhelm Leibniz Universitat Hannover

[CR18] Keech D (2017). Social enterprises with environmental objectives: saving traditional orchards in England and Germany. T Geogr J.

[CR19] Khai HV, Yabe M (2015). Consumer preferences for agricultural products considering the value of biodiversity conservation in the Mekong Delta. Vietnam J Nat Conserv.

[CR20] Klink J, Langen N, Hecht S, Hartmann M (2014). Sustainability as sales argument in the fruit juice industry? An analysis of on-product communication. J Food Syst Dyn.

[CR21] Krueger RA, Casey M (2015). Focus groups: a practical guide for applied research.

[CR22] Langen N (2013). Ethics in consumer choice. An empirical analysis based on the example of coffee.

[CR61] Lee H (2019). Understanding ethical consumers through person/thing orientation approach. J Bus Ethics.

[CR23] Lee H, Hwang J (2016). The driving role of consumers’ perceived credence attributes in organic food purchase decisions: a comparison of two groups of consumers. Food Qual Pref.

[CR24] Lindenberg S, Steg L (2007). Normative, gain and hedonic goal frames guiding environmental behavior Normative. J Soc Issues.

[CR25] Lobe B, Morgan D, Hoffman KA (2020). Qualitative data collection in an era of social distancing. J Qual Methods.

[CR26] Lusk BC, Briggeman JL (2009). Food Values. Am J Agric Econ.

[CR27] Mazzocchi C, Ruggeri G, Corsi S (2019). Consumers’ preferences for biodiversity in vineyards: a choice experiment on wine. Wine Econ Policy.

[CR28] Meier BP, Dillard AJ, Lappas CM (2019). Naturally better? A review of the natural-is-better bias. Soc Personal Psychol Compass.

[CR29] Miele M, Evans A (2010). When foods become animals: ruminations on ethics and responsibility in care-full practices of consumption. Ethic Pl Environ.

[CR30] Moreno G, Aviron S, Berg S, Crous-Duran J, Franca A, de Jalón SG, Hartel T, Mirck J, Pantera A, Palma JHN, Paulo JA, Re GA, Sanna F, Thenail C, Varga A, Viaud V, Burgess PJ (2018). Agroforestry systems of high nature and cultural value in Europe: provision of commercial goods and other ecosystem services. Agrofor Syst.

[CR31] Morgan DL (1997). Focus groups as qualitative research.

[CR32] Mosquera-Losada MR, McAdam JH, Romero-Franco R, Santiago-Freijanes JJ, Rigueiro-Rodríguez A, Rigueiro-Rodríguez MR, McAdam JH, Mosquera-Losada MR (2012). Past, present and future of agroforestry systems in Europe. Advances in agroforestry. Agroforestry—the future of global land use.

[CR33] NABU (Naturschutzbund Deutschland (2018) Studie zur Aufpreisvermarktung von Streuobstprodukten in Baden-Württemberg. MLR, Stuttgart. https://www.km-bw.de/pb/site/pbs-bw-new/get/documents/MLR.LEL/PB5Documents/mlr/Streuobst/2018-08-02%20B1_Kurzfassung%20Streuobststudie_web.pdf. Accessed 8 August 2020

[CR34] Naspetti S, Zanoli R (2021). Consumer perception of sustainable practices in dairy production. Agric Food Econ.

[CR35] Newholm D, Shaw T (2007). Studying the ethical consumer: a review of research. J Consum Behav.

[CR36] Otter V, Langenberg J (2019). Willingness to pay for environmental effects of agroforestry systems: a PLS-model of the contingent evaluation from German taxpayers’ perspective. Agrofor Syst.

[CR37] Pantera A, Burgess PJ, Losada RM, Moreno G, Corroyer N, Mcadam J (2018). Agroforestry for high value tree systems in Europe. Agrofor Syst.

[CR38] Plieninger T, Bieling C, Ohnesorge B, Schaich H, Schleyer C, Wolff F (2013). Exploring futures of ecosystem services in cultural landscapes through participatory scenario development in the Swabian Alb. Germany Ecol Soc.

[CR39] Plieninger T, Levers C, Mantel M, Costa A, Schaich H, Kuemmerle T (2015). Patterns and drivers of scattered tree loss in agricultural landscapes: orchard meadows in Germany (1968–2009). PLoS ONE.

[CR40] Plieninger T, Muñoz-Rojas J, Buck LE, Scherr SJ (2020). Agroforestry for sustainable landscape management. Sustain Sci.

[CR41] Román S, Sánchez-Siles LM, Siegrist M (2017). The importance of food naturalness for consumers: Results of a systematic review. Trend Food Sci Technol.

[CR42] Schaack B, Rampold D, Rogge C (2022). Markt Bilanz Öko Landbau.

[CR43] Schaffner D, Demarmels S, Juettner U (2015). Promoting biodiversity: do consumers prefer feelings, facts, advice or appeals?. J Consum Mark.

[CR44] Schmid O, Dahlke A, Hamm U, Richter T (2005). A guide to successful organic marketing initiatives. Organic marketing initiatives and rural development.

[CR45] Siegrist M (2008). Factors influencing public acceptance of innovative food technologies and products. Trend Food Sci Technol.

[CR46] Siegrist M, Hartmann C (2020). Consumer acceptance of novel food technologies. Nat Food.

[CR47] Sollen-Norrlin M, Gahley B, Rintoul N (2020). Agroforestry benefits and challenges for adoption in Europe and beyond. Sustain.

[CR48] Solomon G, Hogg M, Askegaard M, Bamossy S (2019). Consumer behaviour: a European perspective.

[CR49] Stampa E, Zander K (2022). Backing biodiversity? German consumers’ views on a multi-leve biodiversity-labeling scheme for beef from grazing-based production systems.. J Clean Prod.

[CR59] Tojnko S, Rozman Č, Unuk T, Pažek K, Pamič S (2011). A qualitative multi-attribute model for the multifunctional assessment of “streuobst stands” in NE Slovenia. Erwerbs-Obstbau.

[CR50] Torralba T, Fagerholm M, Burgess N, Moreno PJ, Plieninger T (2016). Do European agroforestry systems enhance biodiversity and ecosystem services? A meta-analysis. Agric Ecosyst Environ.

[CR51] Tsonkova P, Mirck J, Böhm C, Fütz B (2018). Addressing farmer-perceptions and legal constraints to promote agroforestry in Germany. Agrofor Syst.

[CR60] Tu VH, Kopp SW, Trang NT, PaKontoleonek A, Yabeč M (2021). UK consumers’ preferences for ethical attributes of floating rice: implications for environmentally friendly agriculture in Vietnam. Sustain.

[CR52] Vargas AM, de Moura AP, Deliza R, Cunha LM (2021). The role of local seasonal foods in enhancing sustainable food consumption: a systematic literature review. Foods.

[CR53] Vermeir I, Verbeke W (2006). Sustainable food consumption: exploring the consumer ‘attitude—behavioral intention’ gap. J Agric Environ Ethic.

[CR54] Weible D, Christoph-Schulze I, Salamon D, Zander K (2016). Citizens’ perception of modern pig production in Germany: a mixed method research approach. Brit Food J.

[CR55] Wolpert F, Quintas-Soriano C, Plieninger T (2020). Exploring land-use histories of tree-crop landscapes: a cross-site comparison in the Mediterranean Basin. Sustain Sci.

[CR56] Zander K, Hamm U (2010). Consumer preferences for additional ethical attributes of organic food. Food Qual Pref.

[CR57] Zander K, Waibel H (2005). Die Zahlungsbereitschaft für traditionelle Formen der Landbewirtschaftung: das Beispiel ‘Streuobst’. J Environ Law Pol.

[CR58] Zander K, Stolz H, Hamm U (2013). Promising ethical arguments for product differentiation in the organic food sector. A mixed methods research approach. Appetite.

